# High expression of HMGB1 in children with refractory *Mycoplasma pneumoniae* pneumonia

**DOI:** 10.1186/s12879-018-3346-8

**Published:** 2018-08-29

**Authors:** Ying Ding, Chu Chu, Yuqin Li, Gen Li, Xiaoli Lei, Weifang Zhou, Zhengrong Chen

**Affiliations:** 1 Department of Infectious Disease, Children’s Hospital of Soochow University, Suzhou, 215003 China; 2 Department of Respiratory Disease, Children’s Hospital of Soochow University, Suzhou, 215003 China

**Keywords:** HMGB1, *M. Pneumoniae pneumonia*, Lipid-associated membrane proteins (LAMPs), RMPP

## Abstract

**Background:**

Increasing numbers of refractory or severe, even fatal, cases of *Mycoplasma pneumoniae* infections have been reported in recent years. Excessive inflammatory responses play a vital role in the pathogenesis of refractory *M. pneumoniae* pneumonia (RMPP). HMGB1 is an actively secreted cytokine produced by macrophages and other inflammatory cells that participates in various infectious diseases. The present study aimed to explore the role and clinical significance of HMGB1 in children with RMPP and the potential mechanism of HMGB1 expression.

**Methods:**

Four hundred and fifty-two children diagnosed with *M. pneumoniae* pneumonia, including 108 children with RMPP, were enrolled from January 2013 to December 2015 at the Children’s Hospital of Soochow University. HMGB1, TNF-α, and IL-6 in peripheral blood from RMPP and non-RMPP (NRMPP) cases were detected by real-time PCR and ELISA. Lipid-associated membrane proteins (LAMPs) were extracted from live *M. pneumoniae* and prepared at different concentrations for stimulation of THP-1 cells. After coculture with LAMPs, HMGB1, TNF-α, IL-6, RAGE, TLR2, and TLR4 in THP-1 cells were detected by real-time PCR.

**Results:**

Occurrences of cough, fever, and abnormal lung signs were more frequent in RMPP cases compared with NRMPP cases (all *p* < 0.05). Children with RMPP had longer hospital stays than children with NRMPP (*p* < 0.05). Different distributions of lymphocytes were noted between RMPP and NRMPP cases. HMGB1, TNF-α, and IL-6 levels were significantly higher in RMPP cases compared with NRMPP cases (all *p* < 0.05). HMGB1 had good diagnostic ability to differentiate RMPP with AUC of 0.876, sensitivity of 0.833, and specificity of 0.824 compared with TNF-α and IL-6. HMGB1 expression in THP-1 cells was increased by stimulation with 10 μg/ml LAMPs. TLR2 expression was increased after stimulation with 6 μg/ml LAMPs. HMGB1 level was positively associated with TNF-α, IL-6, and TLR2 levels.

**Conclusions:**

HMGB1 is a good diagnostic biomarker for differentiating RMPP and NRMPP. LAMPs from *M. pneumoniae* may induce HMGB1 expression in immune cells through the TLR2 pathway. Further in vitro and in vivo studies are needed for the development of a new treatment strategy to inhibit the HMGB1 pathway, thereby preventing the inflammation in RMPP.

## Background

Pneumonia is one of the most serious infectious diseases, with high morbidity and mortality. In 2015, pneumonia killed an estimated 922,000 children under 5 years of age, accounting for 15% of all child deaths. *Mycoplasma pneumoniae* is a major bacterial pathogen of the airways that causes acute and chronic infections of the respiratory tract and accounts for approximately 10–40% of all lower respiratory tract infections including community-acquired pneumonia (CAP) [1, 2]. Children under 5 years of age usually suffer from mild upper respiratory symptoms, while older children and adolescents develop bronchopneumonia and require hospitalization [3].

Several mechanisms, including intracellular localization, direct cytotoxicity, and inflammation response activation through Toll-like receptors (TLRs) leading to pro-inflammatory cytokine-mediated tissue injury, play essential roles in the pathogenesis of pneumonia [4–6]. The symptoms of *M. pneumoniae* pneumonia (MPP) are correlated with the induction of pro-inflammatory cytokines [7]. Excessive inflammatory responses induced by *M. pneumoniae* play vital roles in the pathogenesis of CAP including refractory *M. pneumoniae* pneumonia (RMPP) [8].

RMPP presents with clinical and radiological deterioration despite macrolide antibiotic therapy for ≥7 days [9], and can develop into severe life-threatening pneumonia. IL-10 and IFN-γ may have important roles in RMPP [10].

The mechanism for the excessive inflammatory responses in RMPP has not been fully elucidated. High-mobility group box protein 1 (HMGB1) is an actively secreted cytokine produced by macrophages and other inflammatory cells during innate immune responses to invasion [11]. Like other members of the pro-inflammatory cytokine family, biologically active HMGB1 can be expressed on the plasma membrane or released by activated inflammatory cells to accumulate in vivo during infection. HMGB1 is related to epithelial barrier destruction, organ dysfunction, and even death through its activation of immunocompetent cells to produce some pro-inflammatory factors (such as TNF-α, IL-1) [12]. A previous study demonstrated that surfactant protein-A can inhibit mycoplasma-induced dendritic cell maturation through regulation of HMGB1 cytokine activity to minimize lung inflammation [13]. Thus, HMGB1 may play a role in the inflammatory responses during *M. pneumoniae* infection. To our knowledge, there are no reports on HMGB1 expression in children with RMPP.

The present study aimed to explore the role and clinical significance of HMGB1 in children with RMPP or non-RMPP (NRMPP) and the potential mechanism of HMGB1 expression, and to provide new clues for MPP prevention and treatment.

## Methods

### Patients

Children diagnosed with CAP were enrolled from January 2013 to December 2015 in the Children’s Hospital of Soochow University. The age range was 1 month to 14 years. All patients underwent chest radiography and *M. pneumoniae* tests including specific IgM in peripheral blood by ELISA and specific DNA fragments in nasopharyngeal aspirates by real-time PCR. The exclusion criteria for the study were: (1) patients with congenital heart diseases, hereditary metabolic diseases, neurological disorders, bronchopulmonary dysplasia, and immunodeficiency; (2) patients co-infected with other pathogens.

Finally, a total of 452 hospitalized children with MPP on the basis of either specific IgM in a blood test or positive PCR result combined with chest radiography and physical examination [14] were enrolled during the study period. All of the chest radiographs were reviewed by an experienced radiologist. Data for clinical manifestations and laboratory tests during the disease course were obtained. RMPP cases were defined as those showing clinical and radiological deterioration despite appropriate antibiotic therapy for ≥7 days [9]. Twenty-four children who underwent selective surgery were chosen as healthy controls. The study was approved by the Institutional Review Board of the Children’s Hospital of Soochow University.

### Serology of *M. pneumoniae*

Specific antibodies against *M. pneumoniae* (IgM and IgG) were detected in serum samples from the children during the acute phase (upon admission) and convalescent phase (upon discharge) using a commercial ELISA kit (Shenzen YHLO Biotech, Shenzhen, China) according to the manufacturer’s instructions. Acute *M. pneumoniae* infection was defined as either a single positive serum IgM titer (cut-off 1.1 S/CO) or a 4-fold increase in IgG titer (cut-off 24 RU/mL) in the convalescent serum sample.

### Real-time PCR for *M. pneumoniae* detection

A real-time PCR procedure (Daan Gene Co. Ltd., Guangzhou, China), which was approved by the State Food and Drug Administration of China, was used to detect *M*. *pneumoniae* in real time [15]. Briefly, one sample of nasopharyngeal aspirate was shaken for 30 s and centrifuged at 15,000×*g* for 5 min. The sediment was collected and DNA was extracted from a 400-μl aliquot in accordance with the manufacturer’s instructions. PCR amplification was performed using primers and probes purchased from Daan Gene Co. Ltd. in a 7600 real-time PCR system (Applied Biosystems, Foster City, CA, USA). The PCR conditions were as follows: 93 °C for 2 min; 10 cycles of 93 °C for 45 s and 55 °C for 60 s; 30 cycles of 93 °C for 30 s and 55 °C for 45 s. Quantitative curves were drawn with standard control samples at several concentrations. *M. pneumoniae*-positive samples were defined as those with DNA concentrations exceeding 2.5 × 10^3^ copies/ml to exclude *M. pneumoniae* colonization.

### Detection of TNF-α and IL-6 by ELISA

Peripheral blood was collected on admission and the plasma supernatant was stored at − 70 °C after centrifugation. TNF-α and IL-6 ELISA kits were purchased from Multi Science Company. All procedures were conducted according to the manufacturer’s instructions.

### Preparation of lipid-associated membrane proteins (LAMPs) from *M. pneumoniae*

*M. pneumoniae* strain M129 was purchased from the Institute of Pathogen Biology (Medical College of University of South China). *M. pneumoniae* was grown in SP4 broth for 72 h at 37 °C, centrifuged at 10,000×*g* for 20 min, resuspended in saline to yield 1 × 10^8^ CFU/50 μl, and frozen at − 80 °C until extraction of LAMPs. Briefly, the *M. pneumoniae* pellet was suspended in Tris-buffered saline containing 1 mM EDTA (TBSE), solubilized by addition of Triton X-114 to a final concentration of 2%, and incubated at 4 °C for 1 h. The lysate was then incubated at 37 °C for 10 min for phase separation. After centrifugation at 10,000×*g* for 20 min, the upper aqueous phase was removed and replaced with the same volume of TBSE. The phase separation procedure was repeated twice. The final Triton X-114 phase was resuspended in TBSE to the original volume, and 2.5 volumes of ethanol were added to precipitate membrane components after overnight incubation at − 20 °C. After centrifugation, the pellet was suspended in phosphate-buffered saline and sonicated for 30 s at output 5 (Sonics, USA). The protein concentration of the final suspension was measured with the Coomassie Protein Assay Regent (Pierce, Rockford, IL, USA).

### THP-1 cell stimulation by LAMPs in vitro

The human monocytic cell line THP-1 was purchased from ATCC (Manassas, VA, USA). THP-1 cells were cultured in RPMI 1640 medium containing 10% fetal calf serum (Wisent, Nanjing, China), 2 mM l-glutamine, 100 U/ml penicillin G, and 100 μg/ml streptomycin. The cell density was adjusted to 1 × 10^6^ cells/ml in 24-well plates. The cells were then cultured with different doses of LAMPs (0, 2, 4, 6, 8, and 10 μg/ml) for 16 h. The cells were harvested and immediately stored at − 80 °C until further analysis.

### HMGB1, TNF-α, IL-6, RAGE, TLR2, and TLR4 detection by real-time PCR

Peripheral blood mononuclear cells from children with *M. pneumoniae* infection or freshly isolated THP-1 cells in culture were collected and lysed with Trizol (Invitrogen, Carlsbad, CA, USA). Total RNA was extracted using an RNeasy Mini Kit (Qiagen, Valencia, CA, USA). A total mixture of 15 μl containing 2 μg RNA, 25 μM random hexamers (Sangon Biotech, Shanghai, China), and RNase-free water was prepared for cDNA synthesis at 37 °C according to the manufacturer’s instructions. PCR amplifications were performed in a volume of 20 μl containing 1 μl cDNA, 10 μl primers, and probes (Table 1). The PCR conditions were as follows: 40 cycles of 95 °C for 30 s, 95 °C for 3 s, and 57 °C for 20 s. The gene expression was assessed by the comparative cycle threshold (Ct) method. The relative amounts of mRNA for the TLRs were determined by subtracting the Ct values for these genes from the Ct value for the housekeeping gene GAPDH (ΔCt). The amounts of mRNA were expressed relative to the amount of GAPDH mRNA (2^-ΔΔCt^) and presented as means ± SEM.

**Table 1:** Foward and reverse primers using for real-time PCRs

	Foward (5′ → 3′)	Reverse (5′ → 3′)
TNF-α	CAACCTCTTCTGGCTCAA	TGGTGGTCTTGTTGCTTA
HMGB1	TGTAAGGCTGTGTAAGATT	AAGGTTAGTGGCTATTGAA
IL-6	ACCTCAGATTGTTGTTGTT	TAGTGTCCTAACGCTCATA
RAGE	CAATGAACAGGAATGGAA	AGAGGCAGAATCTACAAT
TLR2	TGAGGAACTTGAGATTGAT	CACGGAACTTGTAACATC
TLR4	TCAGTGTGCTTGTAGTAT	CCTGGCTTGAGTAGATAA
GAPDH	CTCTGGTAAAGTGGATATTGT	GGTGGAATCATATTGGAACA

*HMGB1* High Mobility Group Box Protein 1, *TNF* tumor necrosis factor, *IL* interleukin, *RAGE* receptor for advanced glycation endproducts, *TLR* toll-like receptors, *GAPDH* glyceraldehyde phosphate dehydrogenase

### Statistical analysis

Continuous variables were compared by Student’s *t*-test or the Mann–Whitney *U* test if the clinical or laboratory data had a non-normal distribution. Categorical data were analyzed by the chi-square test. For in vitro studies, statistical significance was determined by one-way ANOVA, followed by the Tukey–Kramer test. Correlations between HMGB1 and other inflammatory factors were evaluated by Spearman’s rank correlation. Data were expressed as means ± SD if data had a normal distribution.

## Results

### Demographic data and clinical characteristics in children with RMPP or NRMPP

In total, 108 cases were defined as RMPP and 344 cases were NRMPP. As shown in Table 2, the RMPP cases had a higher mean age (62.55 ± 35.05 months) than the NRMPP cases (35.78 ± 27.38 months). The occurrences of cough, fever, and abnormal lung signs were more frequent in RMPP cases compared with NRMPP cases (all *p* < 0.05). However, wheezing was more common in NRMPP cases compared with RMPP cases (*p* < 0.05). Children with RMPP had longer hospital stays than children with NRMPP (*p* < 0.05).

**Table 2:** Comparison of demographic data and clinical characteristics between RMPP and NRMPP

	NRMPP (*n* = 344)	RMPP (*n* = 108)	*p* value
Age (month)	35.78 ± 27.38	62.55 ± 35.05	< 0.001
Male (%)	198 (57.6)	54 (50)	0.185
Clinical profiles			
Cough (%)	311 (90.4)	108 (100)	< 0.001
Wheezing (%)	120 (34.9)	14 (13.0)	< 0.001
Nose running (%)	121 (35.2)	40 (37.0)	0.731
Fever (day)	3.39 ± 2.47	9.18 ± 3.55	< 0.001
Difficult feeding (%)	12 (3.5)	2 (1.9)	0.530
Diarrhea (%)	31 (9.0)	9 (8.3)	1.000
Abnormal signs of lungs (%)	169 (49.1)	81 (75.0)	< 0.001
Hospital stay (day)	5.97 ± 0.99	11.04 ± 2.72	< 0.001

*RMPP* refractory *Mycoplasma pneumoniae* pneumonia, *NRMPP* non-refractory *Mycoplasma pneumoniae* pneumonia

The laboratory test data for children with RMPP and NRMPP are shown in Table 3. Total white blood cells, absolute neutrophils, C-reactive protein, IgA, IgG, and IgM in peripheral blood were significantly higher in RMPP cases compared with NRMPP cases. High LDH and CK levels were more frequent in children with RMPP. Different distributions of lymphocytes were noted between RMPP and NRMPP cases (Table 3).

**Table 3:** Comparison of laboratory tests between RMPP and NRMPP cases

	NRMPP (*n* = 344)	RMPP (*n* = 108)	*p* value
WBC(×10^9^/L)	8.67 ± 3.72	10.28 ± 4.33	< 0.001
Absolute neutrophils(× 10^9^/L)	4.20 ± 2.70	6.32 ± 3.59	< 0.001
Platelets(× 10^9^/L)	312.03 ± 109.95	322.65 ± 90.8	0.363
CRP (mg/L)	7.06 ± 7.00	36.08 ± 30.81	< 0.001
High level of LDH(> 382 U/L, %)	164 (47.7)	64 (59.3)	0.153
High level of CK(> 225 U/L, %)	14 (4.1)	21 (19.4)	< 0.001
IgA (g/L)	0.64 ± 0.52	1.25 ± 0.80	< 0.001
IgG(g/L)	7.25 ± 2.36	9.16 ± 3.03	< 0.001
IgM(g/L)	1.28 ± 0.55	1.72 ± 0.82	< 0.001
Lymphocytes distribution			
CD3+ (×10^9^/L)	2.34 ± 1.68	2.00 ± 1.05	0.047
CD3 + CD4+ (× 10^9^/L)	1.36 ± 1.06	1.08 ± 0.64	0.008
CD3-CD8+ (×10^9^/L)	0.82 ± 0.65	0.82 ± 0.44	0.990
CD4/CD8	1.74 ± 0.73	1.35 ± 0.43	< 0.001
CD3-CD19+ (×10^9^/L)	0.97 ± 0.85	0.66 ± 0.56	< 0.001
CD3-CD(16 + 56+) (×10^9^/L)	0.44 ± 0.36	0.37 ± 0.26	0.047
CD19 + CD23 + (×10^9^/L)	0.51 ± 0.48	0.34 ± 0.31	0.001

*RMPP* refractory *Mycoplasma pneumoniae* pneumonia, *NRMPP* non-refractory *Mycoplasma pneumoniae* pneumonia, *CRP* C-reative protein, *LDH* lactate dehydrogenase, *CK* creatine kinase, *CD* cluster of differentiation

### Comparison of HMGB1, TNF-α, and IL-6 levels between RMPP and NRMPP cases

**Fig. 1:**
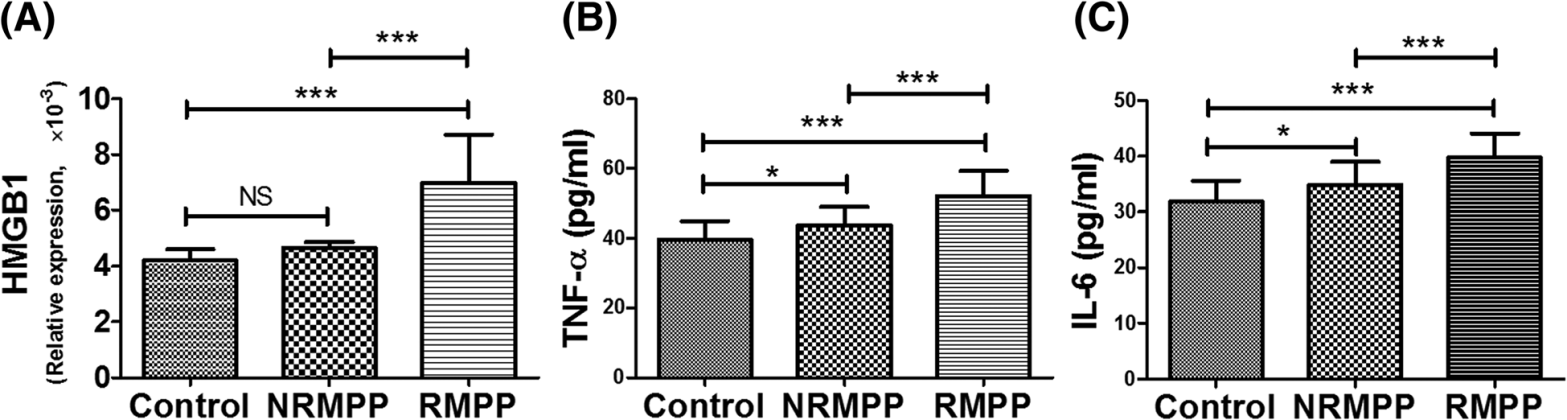
HMGB1, TNF-α, IL-6 levels in children with RMPP or NRMPP. (**a**) Comparison of HMGB1 between RMPP and NRMPP; (**b**) Comparison of TNF-α between RMPP and NRMPP; (**c**) Comparison of IL-6 between RMPP and NRMPP. * RMPP: refractory *Mycoplasma pneumoniae* pneumonia; NRMPP: non-refractory *Mycoplasma pneumoniae* pneumonia. * *p* < 0.05; *** *p* < 0.001

As shown in Fig. 1, the median TNF-α concentration was 50.96 pg/ml in RMPP cases and 42.78 pg/ml in NRMPP cases, the median IL-6 concentration was 39.82 pg/ml in RMPP cases and 34.65 pg/ml in NRMPP cases, and the median HMGB1 concentration was 7.44 × 10^− 3^ in RMPP cases and 4.35 × 10^− 3^ in NRMPP cases. The HMGB1, TNF-α, and IL-6 levels were significantly higher in RMPP cases compared with NRMPP cases (all *p* < 0.05). Compared with healthy controls, no differences were found in HMGB1 levers for NRMPP cases, while higher TNF-α and IL-6 levels were found in NRMPP cases.

**Fig. 2:**
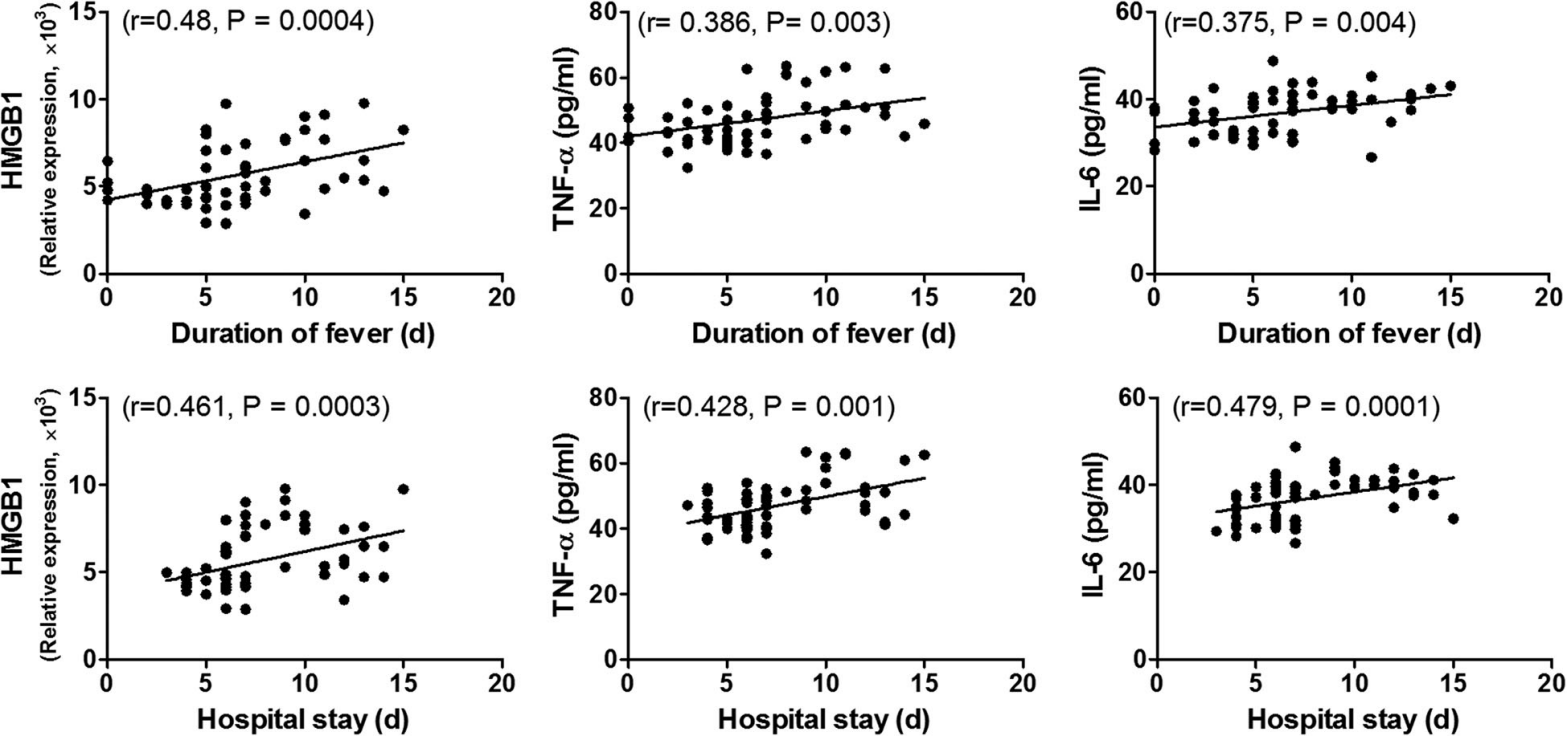
Associations between inflammatory factors including HMGB1, TNF-α, IL-6 and duration of fever and hospital stay. *p* < 0.05 was considered statistically significant

Durations of fever and hospital stay were two major factors associated with disease severity. Thus, we analyzed the correlations between HMGB1, TNF-α, and IL-6 levels and these two major factors. As shown in Fig. 2, HMGB1, TNF-α, and IL-6 levels were positively correlated with duration of fever or hospital stay.

There were interactions among HMGB1, TNF-α, and IL-6. HMGB1 and IL-6 were both correlated with TNF-α (*r* = 0.395, *p* = 0.002 and *r* = 0.268, *p* = 0.042, respectively). Thus, the associations were further analyzed by linear regression using a stepwise procedure to exclude an interaction effect. Only HMGB1 was associated with duration of fever (β = 0.451, *p* = 0.0004), while only TNF-α was associated with duration of hospital stay (β = 0.469, *p* = 0.0002).

### Diagnostic values of HMGB1, TNF-α, and IL-6 in children with RMPP

**Fig. 3:**
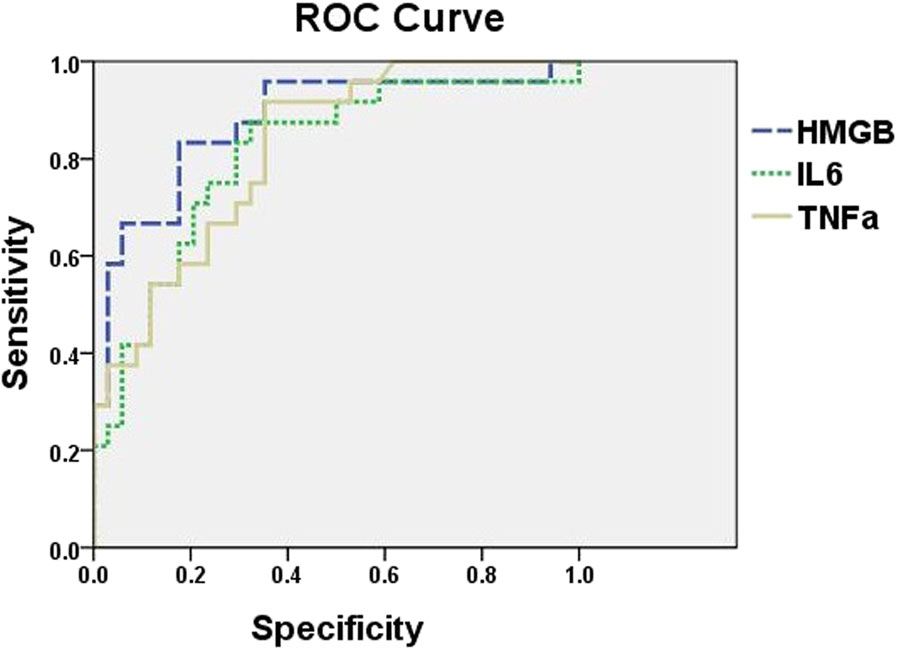
Diagnosis value of HMGB1, TNF-α, IL-6 in children with RMPP. HMGB1 have good diagnostic ability to differentiate RMPP with the AUC of 0.876, the sensitivity of 0.833, and the specificity of 0.824 compared to TNF-α and IL-6

To estimate the diagnostic abilities of HMGB1, TNF-α, and IL-6 in children with RMPP, a receiver-operating characteristic analysis was performed. As shown in Fig. 3, HMGB1 had good diagnostic ability for differentiation of RMPP with cut-off of 5.25 × 10^− 3^, AUC of 0.876, Sig. of AUC of 0.779–0.973, and Youden index of 0.657 compared with TNF-α and IL-6.

### Expression of HMGB1 in THP-1 cells stimulated by LAMPs extracted from *M. pneumoniae*

**Fig. 4:**
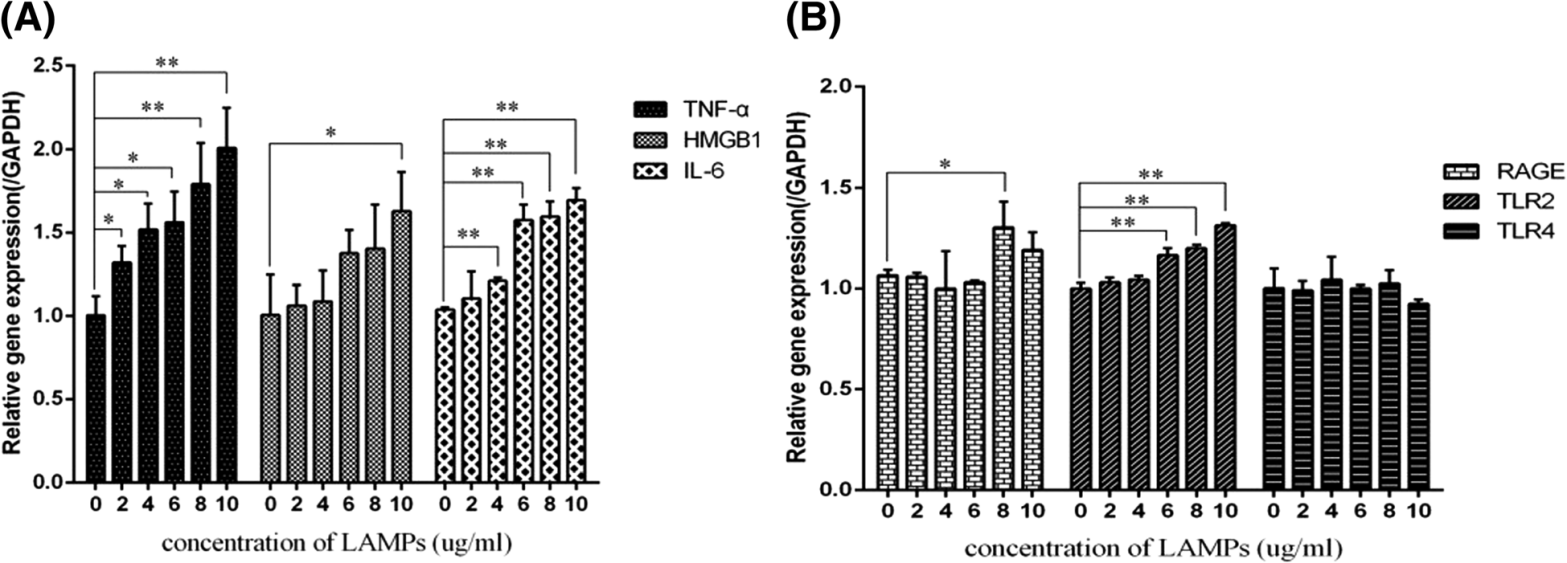
THP-1 cells stimulated by LAMPs extracted from *M. pneumoniae*. THP-1 cells were cultured in RPMI 1640 containing 10% FCS, 2 mM l-glutamine, 100 U/ml penicillin G, and 100 μg/ml streptomycin. The cell concentration of THP-1 was adjusted to 1 × 10^6^ ml in 24-well plates. Different doses of LAMPs (0 μg/ml, 2 μg/ml, 4 μg/ml, 6 μg/ml, 8 μg/ml and 10 μg/ml) were co-cultured with THP-1 cells for 16 h. (**a**) Expression of HMGB1, TNF-α, IL-6 induced by LAMPs; (**b**) Expression of RAGE, TLR2, TLR4 induced by LAMPs. Data are shown as mean ± SEM of 3 independent experiments. * *p* < 0.05; ** *p* < 0.01

In present study, expression of HMGB1 in THP-1 increased after stimulated with 10 μg/ml LAMPs as well as TNF-α with 2 μg/ml and IL-6 with 4 μg/ml. There were also dose-dependent changes in TNF-α and IL-6 expression. RAGE and TLR2 expression was increased after stimulation with 8 μg/ml LAMPs and 6 μg/ml LAMPs, respectively. No difference in TLR4 expression was found after stimulation with LAMPs **(**Fig. 4**)**.

We also analyzed the correlations between HMGB1 and other inflammatory factors in THP-1 cells stimulated by LAMPs. As shown in Table 4, HMGB1 level was positively associated with TNF-α, IL-6, and TLR2 levels.

**Table 4:** Correlations between expression of HMGB1 and other inflammatory factors

Parameters	HMGB1 (relative expression)	
	r	P
TNF-α (pg/ml)	0.7544	0.0003
IL-6 (pg/ml)	0.7709	0.0002
RAGE (relative expression)	0.2755	0.2684
TLR2 (relative expression)	0.6932	0.0014
TLR4 (relative expression)	−0.22303	0.3737

*HMGB1* High Mobility Group Box Protein 1, *TNF* tumor necrosis factor, *IL* interleukin, *RAGE* receptor for advanced glycation endproducts, *TLR* toll-like receptors

## Discussion

Increasing numbers of refractory or severe, even fatal, cases of *M. pneumoniae* infections have been reported in recent years [10, 16], although *M. pneumoniae* infection was traditionally considered to be self-limiting. In the present study, children with RMPP had longer durations of hospital stay and fever as well as high levels of LDH, a useful marker for initiation of steroid therapy [17].

A recent study demonstrated that macrolide-resistant *M. pneumoniae* can lead to clinically refractory pneumonia, showing no clinical or radiological response to macrolides, and further progress to severe and complicated pneumonia [18]. However, the prevalence of macrolide-resistant MPP has rapidly increased, especially in Asian countries, recently reaching 80–90% of cases. In the Suzhou area, the prevalence of macrolide-resistant *M. pneumoniae* accounts for 92.5% of cases (data not shown). Thus, macrolide resistance may be not the main reason for the development of RMPP in children.

Many studies have suggested that excessive inflammatory responses play important roles in RMPP [8, 10]. HMGB1 participates in the pathogenesis of inflammatory diseases and mediates immune responses that range from inflammation and bacterial killing to tissue repair [19]. HMGB1 is associated with divergent clinical conditions such as sepsis [20]. HMGB1 initiates and perpetuates immune responses during infections. A recent study indicated that RSV infection of human airway epithelial cells induces a significant release of HMGB1, which subsequently triggers immune responses by activating primary human monocytes [21]. To our knowledge, there are no reports on HMGB1 expression in children with *M. pneumoniae* infection. We found that children with RMPP had higher HMGB1 levels than children with NRMPP and that HMGB1 had good diagnostic ability to differentiate between RMPP and NRMPP.

TNF-α is mainly produced by monocytes and macrophages, and is a multifunctional peptide with biological activity. TNF-α participates in the lung injury associated with severe pneumonia [22–24]. The TNF-α expression level in children with MPP was significantly higher than that in healthy children. The mechanism is to increase the level of TNF-α in immune cells such as monocytes, macrophages and adhesion receptors after MPP infection,Which is suggested that TNF-α is involved in the pathogenesis of MPP. Wang et al. [8] showed that serum TNF-α levels in children with RMPP were significantly higher than those in children with NRMPP, which was confirmed in a mouse model of MPP infection [25]. The increase in IL-6 was the earliest infection index. IL-6 is an important cytokine in the MPP inflammatory responses. It also has a vital role in the early stage of the immune response [26]. When mycoplasma infection occurs, macrophages produce TNF-α to increase the inflammatory response. By inducing differentiation of T cells, thereby stimulating mononuclear macrophages and lymphocytes to produce IL-6 and other cytokines, the serum concentrations of IL-6 and TNF-α are increased [22]. Studies have shown that cytokines such as IL-6 are associated with the severity of MPP [27].

LAMPs extracted from *M. pneumoniae* can stimulate THP-1 or pulmonary epithelial cells to induce inflammatory responses including production of IL-6, IL-8, and heme oxygenase-1 *in vitro* [28, 29]. Meanwhile, the inflammatory responses induced by *M. pneumoniae* were shown to be associated with pathogen-associated molecular patterns (PAMPs) such as TLR2 and TLR4 [30]. RAGE, a member of the immunoglobulin superfamily of cell surface molecules, is expressed on monocytes and binds to and transduces the signals stimulated by HMGB1. There are no reports on the expression of HMGB1 and its association with other inflammatory factors in THP-1 cells after stimulation with LAMPs. In the present study, it was demonstrated that LAMPs from *M. pneumoniae* could induce HMGB1 expression in THP-1 cells. Meanwhile, HMGB1 was correlated with TNF-α, IL-6, and TLR2. Activation and expression of HMGB1 occur when monocytes or macrophages are exposed to microbe-associated PAMPs such as TLR2 and endogenously-derived inflammatory mediators including TNF-α, IL-1, and IFN-γ [12]. Lipoproteins derived from *M. pneumoniae* can activate NF-κb through TLR2 [31]. Accordingly, we presume that LAMPs enhanced the expression of HMGB1 in THP-1 cells through TLR2. In addition, HMGB1 secreted by monocytes or macrophages can stimulate these cells to release pro-inflammatory cytokines including TNF-α and IL-6 [32]. Further studies are needed to clarify the mechanism of the inflammatory responses induced by HMGB1 .

This article describes a prospective study to investigate the expressions of TNF-α, IL-6, and HMGB1 in children with MPP. TNF-α and IL-6 are pro-inflammatory factors. In previous studies on HMGB1, TNF-α and IL-6 were often used for reference and comparison with HMGB1, especially in studies on sepsis. The study by van Zoelen et al. [33] showed that the serum HMGB1 levels in patients with severe pneumonia were significantly higher than healthy controls, suggesting that HMGB1 is involved in the development of pneumonia. However, HMGB1 has not been reported in patients with MPP. In the literature, HMGB1 was mostly detected by PCR, while ELISA was used by a small number of research groups. The present study is the only research at the cellular level, which is also a deficiency of the study.

## Conclusions

The HMGB1 level in peripheral blood is increased in children with RMPP and is a good diagnostic biomarker for differentiating RMPP and NRMPP. LAMPs from M. pneumoniae may induce HMGB1 expression in immune cells through the TLR2 pathway. Further studies in vitro and in vivo are needed for the development of a new treatment strategy to inhibit the HMGB1 pathway, thereby preventing the inflammation in RMPP.

## Abbreviations

CAP: Community-acquired pneumonia
HMGB1: High-mobility group box protein 1
LAMPs: Lipid-associated membrane proteins
NRMPP: Non refractory Mycoplasma pneumoniae pneumonia
RMPP: Refractory Mycoplasma pneumoniae pneumonia
TLRs: Toll-like receptors
